# The Effects of a Blue-Light Filtering Versus Clear Intraocular Implant on Color Appearance

**DOI:** 10.1167/tvst.10.12.25

**Published:** 2021-10-19

**Authors:** Billy R. Hammond, Billy R. Wooten, Sarah E. Saint, Lisa Renzi-Hammond

**Affiliations:** 1 Vision Sciences Laboratory, Behavioral and Brain Sciences Program, Department of Psychology, University of Georgia, Athens, Georgia, USA; 2 Department of Psychology, Brown University, Providence, Rhode Island, USA; 3 Institute of Gerontology, Department of Health Promotion and Behavior, University of Georgia, Athens, Georgia, USA

**Keywords:** color vision, color appearance, blue-light filter, intraocular lens, cataract

## Abstract

**Purpose:**

More than a dozen studies have investigated whether blue-light filtering (BLF) intraocular lens (IOL) implants influence color vision, generally finding they do not. These studies have not tested color vision per se; rather, they have measured color vision deficiencies or chromatic discrimination. Here, we used additive trichromatic colorimetry to assess color appearance in participants with BLF and clear IOL.

**Methods:**

Seventy-six participants were recruited from two populations: older participants (*n* = 52) with BLF and clear IOL (*n* = 98 eyes; *M* = 67.33 ± 7.48 years; 58.8% female; 25.5% non-White), and young adult control participants (*n* = 24; *M* = 21.0 ± 5.13 years; 70.8% female; 41.5% non-White). Participants used a custom-built tricolorimeter to mix three primaries until a perceived perfect neutral white was achieved. Color appearance, expressed as chromaticity coordinates, was measured with a spectral radiometer (ILS950).

**Results:**

Between subjects, the BLF IOL chromaticity coordinates (*x* = 0.34, *y* = 0.35, u′ = 0.21, v′ = 0.48) were not significantly different from the clear IOL (*x* = 0.34, *y* = 0.33, u′ = 0.22, v′ = 0.48). BLF and clear IOL were also not different within-contralateral subjects (*n* = 21; BLF *x* = 0.34, *y* = 0.33, u′ = 0.22, v′ = 0.47; clear *x* = 0.34, *y* = 0.33, u′ = 0.21, v′ = 0.48). Both IOL groups differed from young adults (v′[0.45; *P* = 0.001], *x*[0.31; *P* = 0.008], and *y*[ 0.30, *P* < 0.000], but not u′[0.21]).

**Conclusions:**

One advantage of geometric representation of color space is the ability to specify the appearance (rather than spectral composition) of any light mixture by specific coordinates. Using this system, only minor differences in color appearance were found between a BLF, clear IOL, and young natural lens.

**Translational Relevance:**

When color perception is directly measured, the BLF and clear IOL are not meaningfully different.

## Introduction

In 1994, Hoya Surgical Optics, based in Singapore, was the first to introduce a yellow-tinted polymethyl methacrylate intraocular lens (IOL) implant. This was followed (circa 2000) by Alcon (Alcon Laboratories, Fort Worth, TX), who also developed a blue-light filtering (BLF) IOL that was promoted on a more global scale. These lenses were originally developed based on a simple premise: existing IOLs did not resemble the healthy adult version of the lens they were replacing. Older, more vulnerable eyes, are actually somewhat protected by (or, at least, are adapted to) a more yellowed lens.^1^ Hence, it seemed logical that implanting a lens tinted to match a normal adult lens represented a more natural prosthesis. Indeed, there were numerous reports around that time that clear IOLs induced significant perceptual changes to color vision.^2^^,^^3^ For example, in an article entitled “Colors do look different after a lens implant!”^4^ the authors noted:

I received a [clear] implant in my right eye, which I hereafter will call the ‘new eye’. The left, or ‘old eye’, did not receive an implant for a year later. What I was unprepared for were the differences in color and appearance of familiar objects between the two eyes.

With the introduction of naturally tinted IOLs, however, the controversy soon shifted to whether actually adding yellow tinting itself introduced significant changes in color perception. A “change,” of course, is only meaningful relative to its comparison. Cataract, for example, likely changes the perception of short-wave light relative to a younger more transparent lens.^5^ The controversy surrounding BLF IOLs was unusual in that medical treatments are often created in an attempt to mimic the natural state as closely as possible. Most BLF IOLs are designed to mimic the lens absorbance of a 30-year-old.^6^ This practice means, of course, that even a BLF IOL represents significantly more short-wave light reaching the older retina when compared with a cataractous lens. Simunovic et al.^7^ expressed the worry that short-wavelength light absorbing IOLs would result in changed color perception as follows:

Because [short-wavelength light absorbing] IOLs influence the spectral quality of light incident on the retina, one of the anticipated deleterious effects of such lenses is on color vision. Compared with conventional UV-absorbing IOLs, [short-wavelength light absorbing] IOLs would be anticipated to effectively decrease the chromaticity difference between warm and cool colors (ie, they should induce a tritan color vision deficiency).

Why the tinting of a BLF IOL would have “deleterious effects,” but the normal yellow of the adult crystalline lens would not, is unclear. Nonetheless, this type of “back and forth controversy”^8^ inspired a wave of studies that assessed whether yellow IOLs (or BLF filters, generally) had negative effects on color vision. These studies^9^^–^^26^ are shown in the Supplementary Data. All of these studies, unfortunately, suffer at least one and often two major limitations. The first is that none of them actually measured color perception per se. The second is that most of them used a clear implanted lens as their normal control (again, a completely transparent crystalline lens is not “normal” in an adult eye).

With respect to the first issue, most studies evaluating color differences used measures based on discriminable differences based on metameric color space, chromatic discrimination, or color vision deficiencies. These studies have shown that BLF IOLs (mostly relative to clear IOLs) do not induce clinical deficits (e.g., analogous to missing a cone type or having anomalous opsins). Subjects with BLF or clear IOLs can also generally perform metameric matching. That is somewhat different, however, to saying that colors appear the same to them as they do to adults with an intact natural lens. For example, one cannot infer appearance (that a particular light looks “vivid blue”) from the color equations of a metameric match or an ordering of chromatic plates.

There are well-validated methods for measuring the actual appearance of colors. One well-studied method is by using the achromatic or white point^27^^,^^28^; that is to say, where the stimuli seem to be totally devoid of chromatic color. The advantage of using the white point is that the zone of normal is very well-documented^29^ and can be quantitatively expressed as CIE chromaticity coordinates (*x* and *y* values). The interpretation is also quite straightforward: the subject's white point is based on their response regarding the appearance of the light (i.e., it looks white and has no chromatic tint); physiologically, that white point is only achieved when the chromatic systems are balanced.

In this study, we used a case control design to assess the effects of a BLF IOL on color appearance. Subjects with the BLF IOL (cases) were compared against two different controls: older subjects with a clear IOL and younger subjects with their natural crystalline lenses. Color appearance was measured using additive trichromatic colorimetry.

## Methods

### Ethics

All study procedures and materials were approved by the University of Georgia Institutional Review Board before initiating the study. All participants gave both written and verbal informed consent before participation, and the tenets of the Declaration of Helsinki were adhered to at all times during the execution of the study.

### Subjects

Seventy-six participants were recruited from two populations: (1) older participants (*n* = 52) from the Northeast Georgia Public Health District (*M* = 67.33 ± 7.48 years; 58.8% female; 25.5% non-White/non-Caucasian), referred by a local ophthalmic practice, who had undergone cataract extraction surgery with IOL replacement in one or both eyes a minimum of 9 weeks before enrollment (*M* = 54 ± 42 months after surgery); and (2) younger participants (*n* = 24; *M* = 21.0 ± 5.13 years; 70.8% female; 41.5% non-White/non-Caucasian) from the larger University of Georgia community.

To be eligible for participation, post-surgical older adult participants must have been implanted with either the clear control lens (Alcon SA60AT), the test BLF lens (Alcon SN60AT), or a combination of the two, one in each eye. Out of the 52 older adult patients recruited (see Table 1 for demographic info on all participants, by test lens), 21 participants had the BLF IOL in one eye and the clear control lens implanted in the other eye and contributed to both a BLF and a clear between-subjects analysis and a separate within-subjects contralateral analysis. The time since implantation was recorded (Table 1) and analyzed (both between subjects and across eyes), but did not influence the overall results. Both older and younger participants were also required to have a minimum of 20:40 (Snellen notation) uncorrected vision or better to participate, which was confirmed upon enrollment. Ishihara pseudoisochromatic plates were used to screen for color anomaly. A small percentage of subjects had detectable trichromatic anomalies but were not excluded (see Table 1). The apparatus (described elsewhere in this article) was created to measure even subtle differences of color appearance. Participants who used spectacle lenses or contact lenses did not use those corrective devices during testing.

**Table 1.** Participant Demographics and Baseline Visual Acuity, by Population

Variables	Subjects	BLF Group	Clear Group
Older			
Age (years)	67.33 ± 7.48	67.28 ± 6.72	67.39 ± 8.28
Gender	58.8% female	64.0% female	53.8% female
Ethnicity	96.1% non-Hispanic	96.0% non-Hispanic	96.2% non-Hispanic
	3.9% Hispanic	4.0% Hispanic	3.8% Hispanic
Race	75.4% White	76.0% White	73.1% White
	19.6% Black	20.0% Black	19.2% Black
	2.0% Asian	0% Asian	3.8% Asian
	3.9% Latinx	4.0% Latinx	3.8% Latinx
Iris color	23.5% light irides	28.0% light irides	19.2% light irides
	49.0% medium irides	48.0% medium irides	50.0% medium irides
	27.5% dark irides	24.0% dark irides	30.8% dark irides
Presence of color anomaly	94.1% no defect	92.0% no defect	96.2% no defect
	5.9% R-G color anomalous	8.0% R-G color anomalous	3.8% R-G color anomalous
Visual acuity (both eyes)	41.2% were 20:20 or better	20.0% were 20:20 or better	61.5% were 20:20 or better
	41.2% were 20:30	60.0% were 20:30	23.1% were 20:30
	17.6% were 20:40	20.0% were 20:40	15.4% were 20:40
Weeks or days between implantation and visual function testing	53.9/3.7 ± 42.0/2.0	42.6/4.0 ± 34.4/1.9	74.4/3.1 ± 47.0/2.1
Younger			
Age (years)	21.0 ± 5.13		
Gender	70.8% female		
Ethnicity	83.3% non-Hispanic		
	16.7% Hispanic		
Race	58.5% White		
	4.0% Black		
	20.8% Asian		
	16.7% Latinx		
Iris Color	12.5% light irides		
	33.33% medium irides		
	54.17% dark irides		
Presence of color anomaly	91.7% no defect		
	8.3% R-G color anomalous		
Visual acuity (both eyes)	62.5% 2 were 0:20 or better		
	8.3% were 20:30		
	29.2% were 20:40		

### Apparatus and Procedure

#### Apparatus

**Figure 1.**
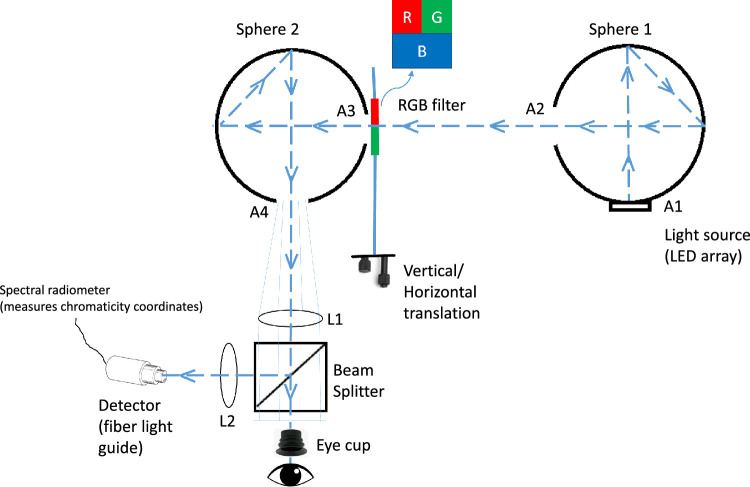
Schematic of the tricolorimeter.

A custom-designed tricolorimeter was constructed to determine and specify the locus of perceptual white within the CIE chromaticiy diagram, as schematized in Figure 1. The optical system was built around two integrating spheres, 1 and 2 as shown in Figure 1. They were constructed from aluminum hemispheres painted on the hollow side with white paint, which is nonluminescent, highly diffusing, and 98% reflecting (Labsphere, North Sutton, NH). Each hemisphere was drilled for two apertures (1-inch diameter, labelled A1-A4). The finished hemispheres were joined with a rigid adhesive to form the two spheres.

The light source was a 1-inch diameter, chip-on-board array of cool white LEDs (6500° color temperature). The array was located at A1 in Sphere 1. The dashed line, originating at the center of the array, traces the path of the principle ray as it traverses through the entire system ultimately entering the eye. From A1, the principle ray projects to the opposite side of the sphere where it is reflected and diffused in all directions. At that point one can follow a principle ray projecting at a 45° angle that impinges at the point that is directly opposite the center of A2. Again, light is diffusively reflected at all angles from every impingement ad infinitum. Thus, the exit port, A2, becomes a Lambertian emitter where the luminance toward an observer is independent of the viewing position; the perception is one devoid of all texture and perfectly uniform (appearing like a disembodied light).

As shown in the Figure 1, the principle ray passes through the center of A2 toward the center of A3 passing into sphere 2. Very near A3 is a filter assembly composed of a blue (B), a green (G), and a red (R) Wratten filter (the important characteristic of each is simply that it provide a highly saturated primary color, in our system the λmax was 447, 543, 615 nm; Wratten filters, #47, #40, #26, respectively; Edmund Optics, Barrington, NJ). The assembly is mounted onto a vertical/horizontal micrometer stage allowing various proportions of the RGB filters to be sampled. A wide ratio of the three colored filters can be set to sample a large subset of the CIE chromaticity diagram ranging from clearly red, or green, or blue to a perfect white, and all shades in between. Several opaque shields were positioned such that the cone of light from A2 was blocked except for the rays entering A3, preventing crosstalk between the various components. After passing through the RGB filter the principle ray impinges on the surface opposite from A3 and follows a path similar to sphere 1, the light exiting at A4. Sphere 2 serves to additively mix the R, G, and B components thoroughly so that the emitted light from A4 is Lambertian as for A2 and color appearance is constant across the perceived target for a given R, G, B setting.

After the principle ray passes through the center of A4, it is transmitted through a lens (L1) and then passes into a beam splitter where half the light is reflected onto a lens (L2) and positioned on the detector of a spectral radiometer, which calculates the chromaticity coordinates. The other one-half of the light is directed through an eyecup and into the eye. L1 is initially positioned one focal length (4 inches) from A4. Thus, the rays emerging from A4 are collimated before passing into the eye where they are imaged on the retina, as shown in the diagram. For an emmetrope A4 would be in sharp focus. But for a myope or a hyperope the image would be in front of the retina or behind the retina, respectively. Perfect focus of the image for any observer was achieved by simply increasing or decreasing the distance between A4 and L1 (one example of a class of telecentric lens assemblies). The primary advantage over other focusing procedures is that the magnification of the image is constant in size no matter the distance between A4 and L1. For our application, the eye cup must be fixed, because it is the reference point for eye position. Therefore, we mounted the Spheres on a platform, which can be translated along the *z*-axis. The observer varies position by turning a dial until perfect focus is achieved.

#### Overview of the Psychophysical Technique

Before the start of each session, the experimenter demonstrated to the subject how turning the knobs changed the color appearance of the test stimulus and explained that the goal was to identify “pure white” (“snow white,” no identifiable tints or colors). The tester then moved the control knobs to achieve a maximal saturation starting point that participants reliably identified as “red.” From that point, the subject verbally guided the experimenter's adjustments until no hint of hue was perceived, with a criterion point of “pure white.” The same procedure (described elsewhere in this article) was repeated for maximal saturation starting points that participants reliably identified as “red,” “green,” “yellow” and “blue.”

#### Finding “White”

From each of these three starting points, the experimenter systematically adjusted one axis (e.g., red–green) at a time and instructed the subject to state when the visual field was as close to white as possible along that axis, at which point the experimenter stopped turning that dial. Then, the experimenter moved to the other axis (red–blue in this example) and adjusted that dial until the visual field was as close to white as possible. This continued, back and forth, with small, fine-tuned adjustments, until the subject reported that the visual field was pure white without any tint of color.

Once an approximate white setting was obtained, subjects were asked to look away from the device for approximately 5 seconds, and then back into the eye piece to double check that the visual field did not contain any tint of color at that setting, by asking questions such as, “If you had to call what you are seeing a color other than white, what would you call it?” Also, using that setting, the experimenter turned each knob to bracket the area (meaning, at what point did the subject first perceive a tint). Four trials, one from each starting point, were collected for each test eye (the order of testing was randomly varied). For the purpose of analysis, we recorded the CIE color space coordinates, *x* and *y*, and u′, v′ (based on the 1976 update to the CIE diagram; the more perceptually Uniform Color Space; for a review see Schanda [2007]^30^). We also recorded total illuminance (lux) of the stimulus, and total irradiance (uW/cm^2^).

## Results

### Between-Subjects Comparison

**Figure 2.**
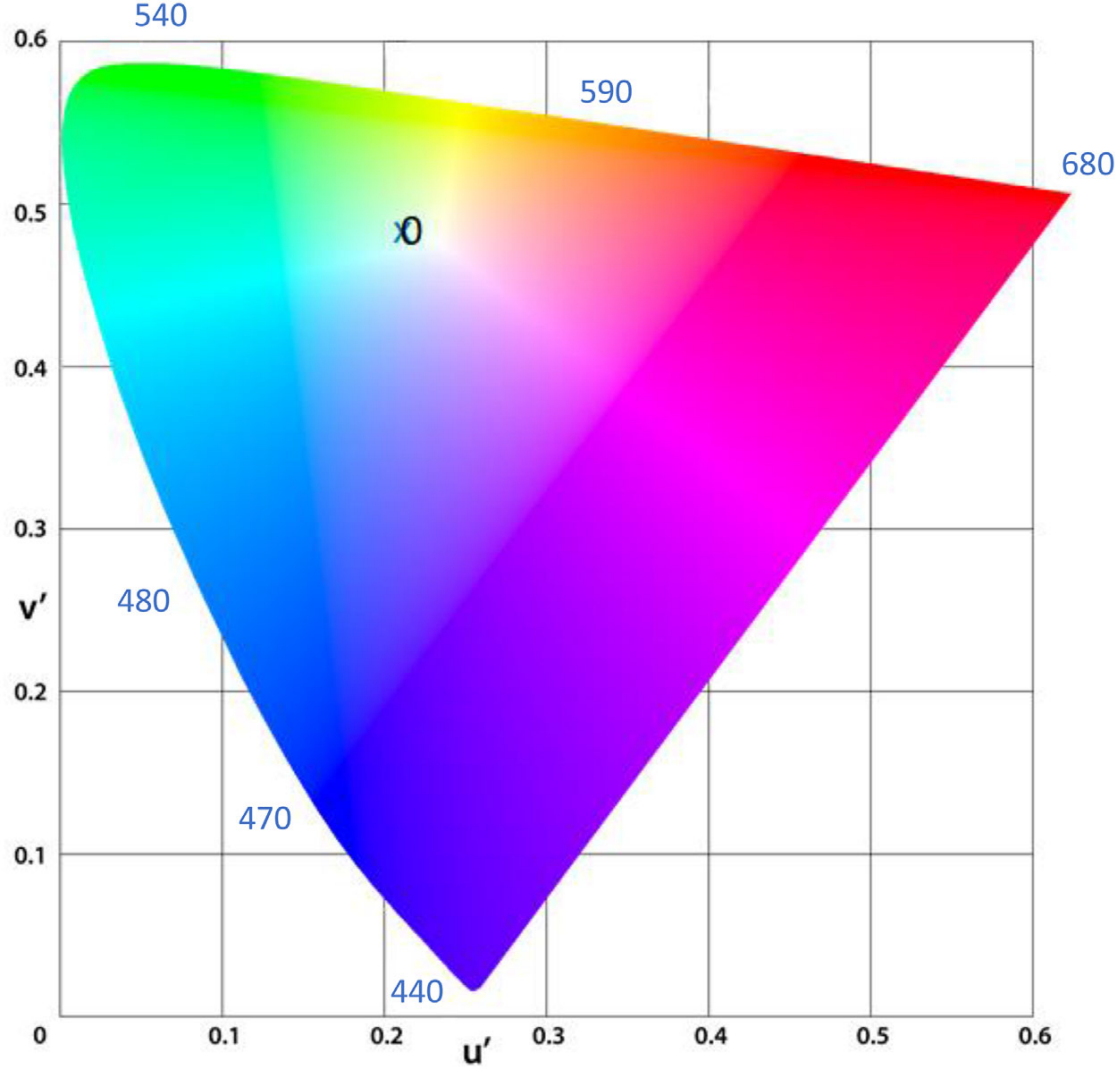
Between group comparison of the chromaticity coordinates of the BLF (*x*) and clear (0)IOL group.

The between-subjects comparison was completed on all 52 IOL participants (26 data points per lens type). A one-way ANOVA was conducted to compare color perception between BLF and clear control participants. There were no significant differences between the BLF group and clear control group on any of the color perception parameters tested, with the exception of total illuminance (*F*[2,47] = 4.004, *P* = 0.03), which was higher in the BLF group (*M* = 152.43 ± 30.32 lux) compared with the clear control group (*M* = 137.77 ± 20.57 lux) (see Table 2 and Fig. 2).

**Table 2.** Color Perception Between Groups

Variable	BLF + Clear IOLs	BLF Group	Clear Group	*F*	*P* Value	Interpretation
u′	0.212 ± 0.02	0.209 ± 0.02	0.215 ± 0.02	0.707	NS	BLF as good as clear
v′	0.476 ± 0.03	0.477 ± 0.03	0.475 ± 0.03	0.066	NS	BLF as good as clear
x	0.338 ± 0.04	0.337 ± 0.04	0.338 ± 0.04	0.017	NS	BLF as good as clear
y	0.339 ± 0.04	0.345 ± 0.04	0.333 ± 0.03	1.131	NS	BLF as good as clear
Total irradiance (µW/cm^2^)	50.94 ± 6.84	52.24 ± 8.10	49.64 ± 5.12	1.829	NS	BLF as good as clear
Total illuminance (lux)	145.10 ± 26.69	152.43 ± 30.32	137.77 ± 20.57	4.004	0.026	Differences due to filtration

NS, not significant.

### Contralateral Comparison

Twenty-one participants had the BLF IOL implanted in one eye, and the clear control lens implanted in the fellow eye (*n* = 42 test eyes). Paired-samples t-tests were used to determine whether there were differences between the eye with the clear lens and the eye with the BLF. There were no significant differences between the eyes on any of color perception parameters measured (see Table 3).

**Table 3.** Contralateral Comparison

Variable	BLF Eye	Clear Eye	Paired Samples *T*	*p*	Interpretation
u′	0.22 ± 0.02	0.21 ± 0.24	0.568	NS	BLF as good as clear
v′	0.47 ± 0.02	0.48 ± 0.04	–0.895	NS	BLF as good as clear
x	0.34 ± 0.03	0.34 ± 0.05	0.306	NS	BLF as good as clear
y	0.33 ± 0.03	0.33 ± 0.03	–0.324	NS	BLF as good as clear
Total irradiance (µW/cm^2^)	49.70 ± 5.19	49.73 ± 5.27	–0.104	NS	BLF as good as clear
Total illuminance (lux)	136.93 ± 19.84	137.84 ± 21.70	–0.369	NS	BLF as good as clear

NS, not significant.

### Comparison With Young Subjects

A one-way analysis of variance was conducted to compare color perception in post-surgical patients against young controls with natural lenses. Color perception in young viewers was significantly different from older viewers (see Table 4) in the following parameters: v′ (*P* < 0.001), *x* (*P* = 0.008), and *y* (*P* < 0.001). Older viewers were otherwise not significantly different from each other on any parameter other than total illuminance, described in the between-subjects analysis above. Older participants with the BLF (*M* = 152.43 ± 30.32) significantly differed (*P* = 0.012) from young participants (*M* = 131.20 ± 28.87) in total illuminance (according to the standard models, the achromatic channel referring to the perception of white and black).

**Table 4.** Age Comparison

Variable	Young Subjects	BLF Group	Clear Group	*F*	*P* Value	Interpretation
u′	0.208 ± 0.01	0.209 ± 0.02	0.215 ± 0.02	0.794	NS	No significant differences between groups
v′	0.452 ± 0.02	0.477 ± 0.03	0.475 ± 0.03	6.578	0.001	Young natural lens is different from both IOL
x	0.311 ± 0.02	0.337 ± 0.04	0.338 ± 0.04	5.122	0.008	Young natural lens is different from both IOL
y	0.303 ± 0.02	0.345 ± 0.04	0.333 ± 0.03	10.116	0.000	Young natural lens is different from both IOL
Total irradiance (µW/cm^2^)	49.60 ± 10.16	52.24 ± 8.10	49.64 ± 5.12	0.875	NS	No significant differences between groups
Total illuminance (lux)	131.30 ± 28.87	152.43 ± 30.32	137.77 ± 20.57	3.991	0.012	BLF IOL is significantly different from young natural lens
						Clear lens is not significantly different from young natural lens

NS, not significant.

### Color Anomaly

A total of 5.9% of older participants and 8.3% of younger participants presented with red–green color anomalous vision. Because the older participants were distributed between the BLF and clear control test groups and approximately equal in number to the young adult comparison group, removing those participants from the analysis did not impact the magnitude, direction, or significance of any of the results significantly.

## Discussion

Alzahrani et al.^31^ recently modelled the effects of commercially available blue-blocking spectacle lenses (BBSL) on color perception. This was done by weighting the transmission characteristics of each lens by a standard spectral sensitivity curve. On this basis the authors concluded that BBSL are “capable of reducing the perception of blue colours … by 5–36 per cent.” It is certainly clear that BBSL filter short-wave light, but do they actually influence the perception of color? One issue with Alzahrani et al.'s modeling is that they used fixed values for spectral sensitivity. Past studies have shown that the visual system can compensate for short-wave filtering by selectively increasing gain in the relevant chromatic system.^32^^,^^33^ Given variable lighting conditions and the high density of the crystalline lens and macular pigment in many individuals, the visual system is optimized to adjust for changes in light incident on the photoreceptors. In a follow-up study (*n* = 5), Alzahrani et al.^34^ used a visual search color detection task where the chromatic content of the target was systematically desaturated (two annuli of small circles were presented for 500 ms and the subject selected the one that contained one circle that was not achromatic). They noted that the spectacle BBSL induced a “‘tritan-like defect’ which affected the perceived color of objects and the contrast detection of object color.” A test that measures the ability to discriminate chromatic vs achromatic stimuli, however, does not address the “perceived color of objects,” nor do the chromatic discrimination tests listed in the Supplementary Data Table.

To assess color appearance, subjects must make a determination regarding the actual perceived color of a stimulus. The question is not trivial. Nocebos (negative expectations leading to a more negative view of the treatment) are common in many areas of medicine (e.g., Barsky et al.^35^); if patients believe that implanting a tinted lens will result in color vision disturbances, the probability that they will perceive changes increases. Patients with a clear implant in one eye and a tinted lens in the other have had explantations (the tinted lens removed) based on perceived alterations in their color perception.^36^ Whether such effects are actual or the result of negative expectations is important to assess.

To this end, we asked subjects (a young healthy comparison group and a sample of older subjects with implants) to assess the whiteness of a stimulus composed of blue, green, and red primaries. Subjects were tested who had BLF IOLs and clear IOLs (both between and within subjects), and their own crystalline lens. We found no differences in the contralateral comparisons (BLF vs clear in the same subject). We also found no differences in color appearance in the between subject comparisons (between IOL and between age). We did see about a 10% increase in illuminance in the BLF group (see Table 2) compared with the clear control group, likely reflecting the simple difference in filtering. We also saw (see Table 4) a slight shift in the white point (*x* values, 8%; *y* values, 11%) when comparing the young vs the older subjects (consistent with past studies^28^^,^^37^^,^^38^).

Taken together, these results support previous work on chromatic discrimination and show that the effects of age and tinting IOLs have minimal effects on color appearance based on the white point. This finding is not surprising because the variation in IOLs is much smaller than individual differences in how much short-wave light is filtered by natural BLFs like the crystalline lens and macular pigment at any age and the fact that the visual system adjusts sensitivity to compensate for large variation in light incident upon the retina. There are some data to suggest that this process takes time. Langina-Jansone et al. (2020)^39^ measured changes in color discrimination directly before and after cataract extraction and did see differences (mostly that inserting a clear lens reduced tritan defects produced by the preceding cataractous lens).

## Supplementary Material

Supplement 1
