# Supplementary material for: The Effects of a Blue-Light Filtering Versus Clear Intraocular Implant on Color Appearance
Source: Transl Vis Sci Technol. 2021 Oct 19;10(12):25. doi: 10.1167/tvst.10.12.25 (PMC8543402; doi:10.1167/tvst.10.12.25)
Supplement: Supplement 1 [file tvst-10-12-25_s001.pdf]

Supplemental Data: Table of past studies that have measured some aspect of color vision in the BLF IOL

Study	Sample	Design	Specifics	Visual test	Result
Ao et al., 2010	43 BLF (mix), 15 Clear	Case-control	1 assess	FM-100 hue test	No difference
Cionni et al., 2006	20 BLF (SN60AT), 17 Phakic	Randomized, prospective case-control	1 assess	FM-100 hue test	No difference
Eberhard et al., 2009	33 subjects, 66 eyes BLF (SN60AT), Clear (SA60AT)	contralateral, Double-masked (randomized eye)	Pre-and post Assess	FM-100	No difference (17% noted color appearance changes)
Greenstein et al. 2007	9 BLF, 9 clear, 9 young phakic	Contralateral,	1 assess	FM-100	No difference
Kara-Junior et al., 2011	30 subjects, 60 eyes BLF (SN60AT), X clear (SA60AT)	Randomized, prospective within-subject	1 assess (5 years post-op)	FM-100 hue test*	No difference
Khokar et al., 2011	50 eyes, BLF (SN60WF), 15 Clear (SA60AT)	Case-control	1, 3 months	Ishihara pseudoisochromatic test, Edridge-Green lantern test, Heidelberg anomaloscope, and Farnsworth-Munsell (FM) 100-hue test	No difference
Landers et al., 2007	90 subjects (pre-existing clear, BLF (SN60AT) implanted	Contralateral (not random, subjects preselected)	Mix of clears	Farnsworth D-15 test, with a subset (*n* = 20) undergoing a Farnsworth–	No difference

				Munsell 100-Hue test	
Leibovitch et al.,	9 BLF (SN60AT), 10 clear (SA60AT)	Randomized, prospective case-control	Tested at 1,3, 6 mos	FM D-15	No difference
Marshall et al., 2005	150 BLF (SN60AT), 147 clear (SA60AT)	Randomized, prospective case-control	1 assess	FM D-15	No difference
Muftuoglu et al., 2007	38 BLF (SN60AT), 38 clear (SA60AT)	Randomized, prospective case-control	1 assess	HMC Anomaloskop MR	No difference
Munoz et al., 2012	28 subjects, 18 eyes BLF (SN60AT), 20 eyes clear (SA60AT)	Non-randomized interventional clinical trial	1 assess	Farnsworth-Munsell (FM) 100-Hue Test under photopic conditions	No difference
Popov et al., 2021	60 subjects, 32 BLF (BioLine Yellow Accurate Aspheric), 28 clear (Tecnis ZcB00)	Case-control	1 assess	Ishihara pseudoisochromatic test and HMC Anomaloskop	No difference
Raj et al., 2005	30 subjects, 60 eyes BLF (SN60AT), Clear (SA60AT)	contralateral, Double-masked (randomized eye)	Pre-and post Assess	Ishihara pseudoisochromatic plates and the Farnsworth D-15 test.	No difference
Rodriguez-Galietero et al., 2005	20 subjects, 40 eyes BLF (SN60AT), Clear (SA60AT)	contralateral, Double-masked (randomized eye)	Pre-and post Assess	FM-100	No difference
Schmidinger et al., 2008	BLF (AF-1 (UY) IOL	Contralateral, randomizied	1 assess	Central and peripheral tritan color contrast	No difference (2 noted color appearance

	(Hoya), and a clear AF-1 UV) in the other*			sensitivities were evaluated using the Moorfields Vision System	changes)
Vuori et al., 2006	19 subjects, 25 eyes BLF (SN60AT) 27 eyes of 18 control patients Clear (SA60AT)	Randomized Double-blind	Pre-and post Assess	FM-100	No difference
Wirtitsch et al., 2009	24 subjects, 48 eyes BLF (SN60AT), Clear (SA60AT)	contralateral, Double-masked (randomized eye)	Pre-and post Assess	Lanthony desaturated D-15 test, the Lanthony new color test (Munsell chroma 2 and 4), HMC anomaloscope.	No difference (3 subjects noted color appearance changes)
Yuan et al., 2004	30 BLF, 30 clear	Randomized, prospective case-control	Tested at 1 week, 1, 3, 6 mos	FM-100	No difference
